# An Electrophysiological Study on the Prevalence of Peripheral Neuropathy in Prediabetics

**DOI:** 10.7759/cureus.104799

**Published:** 2026-03-06

**Authors:** Rammohan G, Thelengana A, Riya Rani Kesh, Riaz B, Mouhamed Nazar, Ilandevan K

**Affiliations:** 1 General Medicine, Sri Venkateshwaraa Medical College Hospital and Research Centre, Ariyur, Pondicherry, IND; 2 General Medicine and Neurology, Sri Venkateshwaraa Medical College Hospital and Research Centre, Ariyur, Puducherry, IND; 3 Ophthalmology, Sri Venkateshwaraa Medical College Hospital and Research Centre, Ariyur, Puducherry, IND; 4 Cardiothoracic and Vascular Surgery, Sri Venkateshwaraa Medical College Hospital and Research Centre, Ariyur, Puducherry, IND; 5 General Medicine, Sri Venkateshwaraa Medical College Hospital and Research Centre, Ariyur, Puducherry, IND

**Keywords:** diabetic neuropathy, glucose intolerance, nerve conduction studies, peripheral neuropathy, prediabetes, sensory and motor nerve conduction

## Abstract

Introduction

Prediabetes is increasingly recognized as a metabolically active state associated with early microvascular complications. Peripheral neuropathy may develop before the onset of overt diabetes, yet its prevalence and electrophysiological characteristics in prediabetes remain insufficiently defined. To estimate the prevalence of clinical and subclinical peripheral neuropathy in individuals with prediabetes using the Modified Toronto Clinical Neuropathy Score (mTCNS) and nerve conduction studies, and to evaluate associated electrophysiological patterns and metabolic correlations.

Methods

This cross-sectional study included 60 individuals with prediabetes diagnosed according to standard glycemic criteria. Participants underwent detailed neurological examination, sensory testing, autonomic assessment, and nerve conduction studies of major motor and sensory nerves. Neuropathy severity was graded using mTCNS. Ocular evaluation was also performed. Statistical analysis assessed associations between neuropathy and metabolic parameters.

Results

The mean age was 46.08 years, and 53.3% were male. Clinical and/or subclinical peripheral neuropathy was identified in 28.4% of participants, with 5% showing severe neuropathy. Nerve conduction studies demonstrated reduced amplitudes, prolonged latencies, and reduced conduction velocities in both motor and sensory nerves. F-wave latencies were significantly prolonged in most nerves. Neuropathy showed significant associations with fasting blood glucose and HbA1c levels, but not with oral glucose tolerance test (OGTT) values, age, or sex. Ocular abnormalities, including cataract, mild diabetic retinopathy, and dry eye disease, were observed in a subset of participants.

Conclusion

Peripheral neuropathy is detectable in a substantial proportion of individuals with prediabetes, even before the onset of overt diabetes. Both clinical and subclinical neuropathy were observed, with significant associations with fasting blood glucose and HbA1c. These findings indicate early neural involvement in dysglycemia; however, causal relationships cannot be inferred due to the cross-sectional design. Larger prospective studies are required to determine the clinical value of early screening and to evaluate whether early intervention can modify long-term neurological outcomes.

## Introduction

Diabetes mellitus, recognized since ancient times, has emerged as one of the fastest-growing global health challenges of the twenty-first century [1-3]. Impaired glucose tolerance (IGT) and impaired fasting glucose (IFG) are well-established metabolic abnormalities that substantially increase the risk of developing diabetes and are collectively categorized as prediabetes [4,5]. The global economic impact of diabetes is substantial, with direct healthcare expenditures projected to reach approximately 1 trillion USD by the year 2030 [6]. Evidence from observational studies indicates that prediabetes is not a benign condition; it is associated with early microvascular and macrovascular complications, including retinopathy, nephropathy, small-fiber neuropathy, and an elevated risk of cardiovascular disease [5]. Importantly, lifestyle interventions have been shown to significantly reduce progression to overt diabetes, achieving relative risk reductions ranging from 40% to 70% among individuals with prediabetes [5].

Diabetic neuropathy represents one of the most common and disabling complications of diabetes, contributing substantially to morbidity, impaired quality of life, and non-traumatic lower-limb amputations worldwide [7]. The prevalence of diabetic neuropathy increases with the duration of diabetes, with reported rates rising from approximately 8% to over 40% within a decade of disease onset [7]. The burden of diabetes and its complications is particularly high in India when compared with Western populations [6]. A systematic review by Trivedi et al. reported that the prevalence of diabetic neuropathy among individuals with diabetes in India ranges between 10.5% and 32.2% [7]. Population-based studies from rural South India have demonstrated even higher prevalence rates, with Darivemula et al. reporting diabetic neuropathy in 39.3% of individuals [8]. Similarly, Gogia et al. documented a prevalence of 41% among patients attending a tertiary care hospital in South India [9]. Despite these observations, longitudinal data evaluating the occurrence and characteristics of peripheral neuropathy during the prediabetic stage remain limited.

Neuropathy in individuals with prediabetes is generally milder than that observed in established diabetes, with sensory involvement occurring more frequently than motor deficits [10]. Small nerve fibers appear to be preferentially affected in early dysglycemic states and may account for the earliest clinical manifestations. The absence of a protective myelin sheath renders these fibers particularly vulnerable to metabolic and osmotic stress induced by hyperglycemia. In contrast, larger myelinated fibers may remain relatively preserved during the initial stages of glucose dysregulation [10]. Data from the Monitoring Trends and Determinants in Cardiovascular Disease (MONICA)/ Cooperative Health Research in the Region of Augsburg, Germany (KORA) study demonstrated a higher prevalence of neuropathy among individuals with IGT compared with normoglycemic controls, with neuropathic pain reported in 13.3% of individuals with diabetes, 8.7% with IGT, 4.2% with IFG, and 1.2% among those with normal glucose levels [11]. Supporting these findings, an Indian study by Gulati et al. reported peripheral neuropathy in 24% of individuals with IFG and 30% of those with IGT when compared with healthy controls [12].

For the purpose of clinical and electrophysiological evaluation, peripheral neuropathy is operationally defined as the presence of neuropathic symptoms and/or signs identified using a validated clinical scoring system, with or without corroborative abnormalities on nerve conduction studies. This definition allows differentiation between clinically evident neuropathy and subclinical neuropathy detected solely through electrophysiological testing.

In addition to neuropathic complications, ocular disorders such as cataracts, diabetic retinopathy, and dry eye disease occur more frequently in individuals with diabetes and may also manifest during the prediabetic stage, preceding the onset of overt diabetic complications [13]. The pathophysiological mechanisms underlying these ocular manifestations overlap with those implicated in peripheral nerve injury and include chronic hyperglycemia, endothelial dysfunction, oxidative stress, and microvascular impairment [14]. Both cataract formation and the progression of diabetic retinopathy have been strongly associated with these shared metabolic and microvascular disturbances [15,16]. Therefore, ocular assessment was included as an exploratory component in this study to evaluate early microvascular involvement that may parallel electrophysiological abnormalities in peripheral nerves.

In this context, the primary objective of the present study was to estimate the prevalence of clinical and subclinical peripheral neuropathy in individuals with prediabetes using the Modified Toronto Clinical Neuropathy Score (mTCNS) and nerve conduction studies.

The secondary objectives were: (1) to characterize the electrophysiological abnormalities observed in motor and sensory nerve conduction parameters, including amplitudes, distal latencies, conduction velocities, and F-wave latencies; (2) to examine the association between neuropathy status and glycemic parameters, specifically fasting blood glucose, oral glucose tolerance test values, and HbA1c; (3) to assess autonomic involvement using sympathetic skin response testing; and (4) to document the frequency of associated ocular abnormalities, including cataract, diabetic retinopathy, and dry eye disease, in the study population.

By clearly defining these outcomes, the study aimed to provide a structured evaluation of early neural and microvascular changes occurring during the prediabetic stage.

## Materials and methods

This is a hospital-based cross-sectional observational study conducted over a period of 18 months, from March 2021 to September 2022, after obtaining approval from the Institutional Ethics Committee (No: 29/SVMCH/IEC-Cert/Feb21). A total of 60 participants aged between 18 and 60 years with a diagnosis of prediabetes were enrolled. Sample size was calculated based on the prevalence of prediabetes, 18%, confidence interval 95%, margin of error 9%, and non-response rate 10%. OpenEpi software version 3.01 (Open-source collaborative project supported by a grant from the Bill and Melinda Gates Foundation to Emory University, Atlanta, USA) was used for the estimation of the proportions. The consecutive sampling method was used for sampling.

Inclusion criteria

Participants were included if they met any one of the following criteria: a) Fasting plasma glucose (FPG) between 100 and 125 mg/dL (5.6-6.9 mmol/L), b) Two-hour plasma glucose between 140 and 199 mg/dL (7.8-11.0 mmol/L) following oral glucose tolerance testing, c) HbA1c between 5.7% and 6.4% (39-46 mmol/mol).

Exclusion criteria

Patients with known diabetes mellitus, macrocytic anemia, malignancy, thyroid disorders, renal insufficiency, or those receiving medications known to cause neuropathy (anti-tubercular drugs, antiretroviral therapy, phenytoin, or steroids) were excluded. Patients with other causes of neuropathy, such as inherited neuropathy, acute or chronic inflammatory demyelinating polyneuropathy, vasculitis, connective tissue disorders, toxin-induced neuropathy, history of limb trauma, alcohol consumption, or critical illness were also excluded.

Clinical assessment

Data were collected using a predesigned structured proforma that included demographic characteristics, symptom profile, treatment history, and outcome indicators. Neuropathy was assessed using the prevalidated mTCNS in addition to nerve conduction studies [17]. All participants underwent a complete general physical and systemic examination, followed by a detailed neurological examination.

Sensory examination

Sensory testing was performed bilaterally with the patient’s eyes closed using standardized tools that included: 1) Pin prick: sharp and dull sensations were tested using a wooden cocktail stick or safety pin applied randomly. 2) Temperature: Two test tubes containing cold water (5-10°C) and warm water (40-45°C) were applied randomly. 3) Light touch: cotton wool or a 1-g monofilament was used. 4) Vibration: A 128-Hz tuning fork was applied to bony prominences. 5) Position sense: joint movements were performed, and participants identified joint position verbally or by mirroring with the opposite limb. Responses were recorded systematically.

Nerve conduction studies

After obtaining informed consent, nerve conduction studies were performed by a certified neurotechnician using the Scorpio-4P NCS system (Allengers Medical Systems Ltd, Chandigarh, India). Motor and sensory studies included: a) posterior tibial motor, b) median motor and sensory, c) ulnar motor and sensory, d) deep peroneal motor, e) superficial peroneal sensory, f) sural sensory, g) sympathetic skin response recording was also performed.

The electrophysiological cut-off values used to define abnormal nerve conduction were based on normative data obtained from healthy individuals in our region and established within our laboratory. These reference values account for regional and laboratory-specific variations and are consistently applied in routine nerve conduction studies.

Subclinical neuropathy is defined as an electrophysiological abnormality of nerve function with no clinical symptoms of peripheral nerve disease.

Ophthalmological evaluation

Each participant underwent a comprehensive ocular examination that included visual acuity (Snellen’s chart), slit lamp examination, dilated fundus examination using indirect ophthalmoscopy with a 20-D lens, dry eye evaluation, dry eye assessment [which included Schirmer’s test, tear break-up time (TBUT), tear meniscus height, and slit lamp evaluation for debris], cataracts were graded using the Lens Opacities Classification System III (LOCS III).

Significant cataract was defined as nuclear opacity ≥4, nuclear color ≥4, cortical cataract ≥2, posterior subcapsular cataract ≥2 [15]. Diabetic retinopathy was classified using the Early Treatment Diabetic Retinopathy Study (ETDRS) scale [16]. Dry eye was classified as a) mild: Schirmer 6-10 mm, TBUT 6-10 s, b) moderate: Schirmer 3-5 mm, c) severe: Schirmer ≤2 mm

A total score ≥4 in either eye was considered positive. Patients with ocular abnormalities were referred for ophthalmologic management.

Statistical analysis

Data were entered into Microsoft Excel and analyzed using SPSS version 23.0 (IBM Corp., Armonk, USA). Quantitative variables, including prevalence of electrophysiological neuropathy and prevalence of clinical and subclinical neuropathy, were expressed as frequencies and proportions. Ocular outcomes were also expressed as frequencies and proportions. Associations between neuropathy status and metabolic variables were analyzed using appropriate statistical tests, and a p-value <0.05 was considered statistically significant.

## Results

The mean age of the study population was 46.08 years with a standard deviation of 9.73 years, 36.6% of the participants were aged between 51 and 60 years, and 53.3% were male. Numbness was the most common symptom reported (96.6%), followed by foot pain and tingling. The most frequent clinical manifestation was abnormal light touch, which was present in 75 percent of the participants. According to the mTCNS, most of them had Grade 1 symptoms, and none of the participants had Grade 3 symptoms. Grade 2 was categorised as foot pain and numbness in 5% and 3.3% of the participants, respectively. In total, 28.4% were found to have neuropathy, with 5% having severe neuropathy, and 71.6% having no signs of neuropathic involvement.

Nerve conduction studies detected 23.3% of the participants with clinical neuropathy and 5% with subclinical neuropathy. Motor nerve examination showed that there was a significant decrease in amplitude of most nerves except the right and left peroneal nerves and the right median nerve at the elbow, where no significant change was observed. The duration of excitation was longer in neuropathic participants, and it was statistically significant in the left median nerve at the wrist and the right peroneal nerve. All the nerves tested, except the peroneal nerves at the ankles and the tibial nerves at the ankles, had a significant increase in motor latency. Also, nerve conduction velocity (NCV) was greatly reduced in all motor nerves in both upper and lower limbs.

Sensory nerve conduction tests showed that there was a significant decrease in sensory nerve action potential (SNAP) amplitudes in the upper limbs and the right sural nerve of the lower limb. The duration of excitation was similar among all the sensory nerves, and no statistically significant differences were found. The latencies of the sensory nerves were greatly increased in all the nerves assessed except the left superficial peroneal nerve. All sensory nerves except the right superficial peroneal nerve showed a significant decrease in NCVs. F-wave analysis showed that there was a significant increase in maximum, mean, and minimum latency values of all the nerves tested except the superficial peroneal nerves in both lower limbs (Table 1).

**Table 1:** Results of Nerve Conduction Study- Distribution of F max, F mean, F min Latency Values in Wave Studies Data were expressed as mean ± standard deviation. Between-group comparisons (neuropathy vs no neuropathy) were performed using independent-samples t-tests. A p-value <0.05 was considered statistically significant.

Nerve tested	Area	F max latency values µ ± SD (ms)		F Mean latency µ ± SD (ms)		Fmin latency values µ ± SD (ms)		p-value		
		Neuropathy	No neuropathy	Neuropathy	No neuropathy	Neuropathy	No neuropathy	F max	F Mean	F min
Left Median N	Upper Limb	32.44±5.35	29.13±2.62	30.78±5.34	27.51±2.40	0.003	0.003	0.003	0.003	0.005
Right Median N		33.72±6.44	29.83±2.93	31.92±5.98	28.01±2.58	0.001	0.001	0.003	0.001	0.001
Left Ulnar N		31.83±5.68	29.39±2.91	30.04±5.26	27.44±2.63	0.016	0.016	0.036	0.016	0.007
Right Ulnar N		32.55±5.80	29.72±2.62	30.71±5.51	27.68±2.28	0.005	0.005	0.014	0.005	0.002
Left Superficial Peroneal N	LowerLimb	53.76±7.95	50.99±5.91	51.83±7.71	48.84±5.45	0.127	0.127	0.183	0.127	0.082
Right Superficial Peroneal N		54.31±6.98	51.27±5.19	52.46±6.52	49.02±4.81	0.066	0.066	0.127	0.066	0.03
Left Sural N		58.57±6.89	52.86±4.87	56.01±6.58	50.48±4.60	0.002	0.002	0.002	0.002	0.002
Right Sural N		59.59±11.04	53.43±5.42	57.11±10.57	50.87±5.04	0.007	0.007	0.012	0.007	0.005

Neuropathy status was not found to have any significant relationship with demographic factors, including age or sex. Conversely, fasting blood glucose and HbA1c showed statistically significant relationships with the presence of neuropathy, but no significant relationship was found between the values of the oral glucose tolerance test (Table 2).

**Table 2:** Association Between Age, Sex, Fasting Blood Sugar, Oral Glucose Tolerance Test, HbA1c, and Neuropathy Among Study Participants Associations were assessed using Pearson’s chi-square test. Fisher’s exact test was applied where expected cell counts were <5. A single test statistic and corresponding p-value are reported per comparison. OGTT: oral glucose tolerance test.

Variable	Category	Neuropathy N (%)	No Neuropathy N (%)	Total	χ² (df)	p-value
Age group (years)	>30	0 (0.00)	4 (100.00)	4	4.31 (3)	0.23
	31–40	4 (30.76)	9 (69.23)	13		
	41–50	4 (19.04)	17 (80.96)	21		
	51–60	9 (40.90)	13 (59.10)	22		
Sex	Male	10 (31.30)	22 (68.80)	32	0.43 (1)	0.51
	Female	7 (25.00)	21 (75.00)	28		
Fasting Blood Sugar	Prediabetic	14 (40.00)	21 (60.00)	35	5.41 (1)	0.02
	Normal	3 (12.00)	22 (88.00)	25		
OGTT	Prediabetic	15 (26.30)	42 (73.70)	57	0.34 (1)	0.56
	Normal	2 (66.70)	1 (33.30)	3		
HbA1c	Prediabetic	12 (40.00)	18 (60.00)	30	4.22 (1)	0.04
	Normal	5 (16.70)	25 (83.30)	30		

In 12 participants (20%), changes in cataracts were reported. Among them, nuclear cataract (NC), cortical cataract (CC), and posterior subcapsular cataract (PSC) were present in two participants each (3.33%). The mixed presentation of NC and CC was observed in three participants (5%), and NC was observed in seven participants (11.67%).

Four participants (6.67%) were found to have diabetic retinopathy. Of them, three participants (5%) had mild non-proliferative diabetic retinopathy (NPDR), and one participant (1.67%) had moderate NPDR. There were no severe NPDR cases, proliferative diabetic retinopathy, or diabetic macular edema.

In nine participants (15%), dry eye disease was diagnosed. Among them, seven participants (11.67%) had mild dry eye, and two participants (3.33%) had moderate dry eye. None of the participants had severe dry eye disease.

## Discussion

Numbness was the most prevalent symptom among the study participants (96.6%), followed by tingling and foot discomfort. Similar findings were reported by Udhayashankar et al. in a study conducted in southern India, in which all participants with diabetes reported tingling, foot discomfort, and numbness [17]. In contrast, Kakrani et al. and Patil et al. observed tingling as the primary complaint in 92% and 97% of their participants, respectively [18,19]. Among the study subjects, the most frequent clinical abnormality was impaired light touch sensation (73.3%), which is comparable to the findings of Udhayashankar et al., who reported light touch abnormalities in 84% of participants.

The mTCNS incorporates sensory testing results and symptom scores to evaluate neuropathy severity on a graded scale from 0 to four. Using this scale, 28.4% of the study participants were identified as having neuropathy, with 5% classified as having severe neuropathy. These findings are consistent with those of Lin et al., who reported a neuropathy prevalence of 29.2% using the Toronto Clinical Neuropathy Scale, with 16.1% and 13.1% of participants having impaired fasting glucose and impaired glucose tolerance, respectively [20].

Motor nerve conduction studies demonstrated a significant reduction in compound muscle action potential amplitudes across most nerves, including bilateral peroneal nerves in the lower limbs and the right median nerve at the elbow. Reduced compound muscle action potential (CMAP) amplitude reflects axonal loss. Vijayalak et al. reported similar reductions in CMAP amplitudes among individuals with impaired glucose tolerance [21]. Comparable findings in the tibial and peroneal nerves were also reported by Rathi et al. [22]. Lal et al. similarly observed reduced CMAP amplitudes in prediabetics with neuropathy [23].

Neuropathic participants exhibited significant prolongation of distal latencies in most nerves, particularly in the peroneal and median nerves. These findings are consistent with those reported by Lal et al. [23]. With the exception of the tibial and peroneal nerves at the ankle, latency prolongation was observed across most examined nerves, in agreement with Agarwal et al. [24]. Nerve conduction velocities were significantly reduced in both upper and lower limb nerves. This slowing may reflect a combination of segmental demyelination, loss of fast-conducting axons, and metabolic alterations within nerve fibers. Similar findings were reported by Rathi et al. and Lal et al. [22,23].

Sensory nerve conduction studies revealed a significant reduction in sensory nerve action potential amplitudes across all upper limb sensory nerves among neuropathic participants, while lower limb sensory involvement was significant only in the right sural nerve. Vijayalak et al. reported similar reductions in sural nerve amplitudes [21]. Rathi et al. also documented comparable sensory nerve abnormalities in neuropathy patients [22].

Latency prolongation was observed in nearly all sensory nerves except the superficial peroneal nerve of the left lower limb. Shariff et al. reported similar latency increases in the sural nerve [25], and Agarwal et al. documented comparable findings [23]. Nerve conduction velocities were significantly reduced in almost all sensory nerves, except the superficial peroneal nerve in the right lower limb. Rathi et al. reported reduced NCV in the sural nerve [22], and Talib et al. observed similar reductions in sensory nerve conduction velocities [26]. In contrast, Mohammad Shoebuddin et al. did not find significant differences in amplitude, latency, or conduction velocity between prediabetics and non-diabetics, which may be attributable to milder neuropathy and a smaller sample size in their cohort [27].

F-wave studies demonstrated significant prolongation of minimum, mean, and maximum latencies in most nerves, except the superficial peroneal nerves bilaterally. Agarwal et al. similarly reported prolonged F-minimum latency in prediabetics with neuropathy [23]. Talib et al. also found prolonged F-wave latencies in prediabetics compared with controls [26]. These findings suggest early proximal nerve involvement in prediabetic neuropathy.

A significant correlation was observed between fasting blood glucose levels and neuropathy status. Majumdar et al. also reported significant metabolic correlations, including abnormalities in glycemic measures, among neuropathy patients in eastern India [28].

No significant association was identified between oral glucose tolerance test (OGTT) values and neuropathy status in the present study. This contrasts with the findings of Lu et al. and Hoffman-Snyder et al., who reported significant associations between OGTT abnormalities and peripheral neuropathy [29,30]. This discrepancy may be due to differences in study populations and the relatively smaller proportion of participants with abnormal OGTT values in the present cohort, which may have reduced statistical power.

HbA1c levels showed a statistically significant association with neuropathy, consistent with the findings of Vijayalak and Kumar, who demonstrated a significant relationship between HbA1c and neuropathy development in prediabetics [21].

Based on clinical scoring and nerve conduction studies, the prevalence of neuropathy in this study was 28.3%, with 23.3% exhibiting clinical neuropathy and 5% showing subclinical neuropathy.

Chronic hyperglycemia, oxidative stress, and small-fiber involvement are known contributors to peripheral nerve injury and microvascular changes. In the present study, ocular abnormalities such as cataract, mild retinopathy, and dry eye disease were observed in a subset of participants. These findings may suggest early microvascular involvement in prediabetes. However, these observations should be interpreted as exploratory. Ophthalmologic evaluation may be considered in selected prediabetic individuals, particularly those with additional risk factors, to allow early identification of ocular involvement. Study limitations: This single-center, cross-sectional study with a small sample size limits generalizability and causal inference. The absence of a normoglycemic control group and the lack of multivariable analysis restrict interpretability. Small subgroup sizes reduce analytical robustness, and ocular findings were exploratory secondary outcomes. Longitudinal progression could not be assessed.

## Conclusions

This hospital-based cross-sectional study identified peripheral neuropathy in a considerable proportion of adults with prediabetes, as assessed by clinical examination and nerve conduction studies. Both clinical and subclinical neuropathic changes were observed. Significant associations were noted between neuropathy and fasting blood glucose as well as HbA1c levels, whereas no meaningful association was found with OGTT values. In addition, a small number of participants showed ocular findings such as early cataract changes, mild non-proliferative diabetic retinopathy, and dry eye disease. These results indicate that neurological abnormalities and selected ocular changes may be present in individuals with prediabetes within the studied group. However, the findings should be interpreted with caution. The study was conducted at a single center with a relatively small sample size and a cross-sectional design, which limits the ability to draw causal inferences. The absence of a normoglycemic control group and limited subgroup sizes further restrict broader interpretation. Therefore, the conclusions drawn from this study apply only to the specific population examined and should not be generalized to all individuals with prediabetes. Larger, multicenter studies with longitudinal follow-up and appropriate comparison groups are needed to better understand the relationship between prediabetes, neuropathy, and early ocular changes.
